# The fundamental role of endothelial cells in hantavirus pathogenesis

**DOI:** 10.3389/fmicb.2014.00727

**Published:** 2014-12-22

**Authors:** Jussi Hepojoki, Antti Vaheri, Tomas Strandin

**Affiliations:** Department of Virology, Haartman Institute, University of HelsinkiHelsinki, Finland

**Keywords:** hantavirus, endothelial dysfunction, HFRS, HCPS, bunyavirus

## Abstract

*Hantavirus,* a genus of rodent- and insectivore-borne viruses in the family *Bunyaviridae*, is a group of emerging zoonotic pathogens. Hantaviruses cause hemorrhagic fever with renal syndrome and hantavirus cardiopulmonary syndrome in man, often with severe consequences. Vascular leakage is evident in severe hantavirus infections, and increased permeability contributes to the pathogenesis. This review summarizes the current knowledge on hantavirus interactions with hematopoietic and endothelial cells, and their effects on the increased vascular permeability.

## INTRODUCTION TO HANTAVIRUSES AND THE ASSOCIATED DISEASE

*Hantavirus* is a genus of rodent- and insectivore-borne (shrews, moles, and bats) viruses of the family *Bunyaviridae*. Hantaviruses cause two human diseases: hemorrhagic fever with renal syndrome (HFRS) in Eurasia and Hantavirus cardiopulmonary syndrome (HCPS) in the Americas. The infection in reservoir hosts is chronic and asymptomatic, and infected animals secrete the virus in their excreta. Thus far only rodent-borne hantaviruses have been associated with human disease. Hantaan (HTNV) and Dobrava–Belgrade hantaviruses (DOBVs) cause a severe form of HFRS (mortality 5–15%) in Asia and Balkans, respectively, whereas Puumala (PUUV) virus cause a milder form of HFRS (mortality <0.1%) in Northern and Central Europe. Seoul virus (SEOV), carried by urban rats, causes a moderate HFRS worldwide. Sin nombre (SNV) and Andes (ANDV) viruses are the main causative agents of HCPS (mortality ∼40%) in Northern and Southern America, respectively. Also non-pathogenic or less virulent rodent-borne hantavirus species such as Prospect Hill (PHV) and Tula (TULV), which both genetically cluster close to PUUV, have been recognized (Jonsson et al., 2010; Vaheri et al., 2011, 2013; Klempa et al., 2013).

The incubation period of HFRS is commonly 2–4 weeks but it may vary from 10 days up to 6 weeks. The course of HFRS is divided into five phases: febrile, hypotensive, oliguric, polyuric, and convalescent. The febrile phase of 3–6 days starts with rapid onset of fever accompanied with myalgia, headache, prostration, thirst, nausea, vomiting, abdominal pain, blurred vision, dizziness, and flushed face. In severe cases fever is followed by decline in blood pressure (the hypotensive phase) that is accompanied by thrombocytopenia, leukocytosis and signs of disseminated intravascular coagulation (DIC). Vascular leakage, commonly occurring at this phase, is manifested as petechiae, periorbital edema, hemoconcentration, and hypotension. The most severe cases may even lead to a fatal shock within 4–5 days after onset of symptoms. Hemorrhages continue with ecchymosis, melena, hematemesis, and epistaxis. Oliguria, hematuria, proteinuria, and polyuria are signs of renal failure and they precede the convalescent phase that may require several weeks (Lee, 1989; Peters et al., 1999; Jonsson et al., 2010). In mild HFRS the phases are not easily distinguished and signs of vascular leakage may be absent. Typically 10% of PUUV- and 30–70% of HTNV-infected patients show hemorrhages (Lahdevirta, 1971; Lee, 1989).

Similarly to HFRS, the incubation period in HCPS is rather long, ranging from 1 to 5 weeks. The course of HCPS is divided into febrile, cardiopulmonary, diuretic, and convalescent phase. The symptoms begin with non-specific febrile phase of 3–5 days that may be include headache, dizziness, nausea, anorexia, diarrhea, and abdominal pain. Pulmonary and/or cardiogenic complications follow the febrile phase. Symptoms of pulmonary edema include dyspnea, tachypnea, and non-productive cough, which are likely due to leakage of lung capillaries. Resulting hypoxia may cause tachycardia and shock, which may be fatal (Peters et al., 1999; Jonsson et al., 2010). The pulmonary edema formed during cardiopulmonary phase is cleared during diuretic phase. Like in HFRS, thrombocytopenia, oliguria, renal failure, and hemorrhages are often diagnosed in HCPS caused by South American viruses such as ANDV but are strikingly often missing in SNV-caused HCPS (Macneil et al., 2011). Due to the fact that some HFRS cases involve pathological findings similar to HCPS cases a common name (hantavirus disease) for both diseases has been suggested (Vaheri et al., 2013).

## MECHANISMS OF ENDOTHELIAL CELL PERMEABILITY IN HANTAVIRUS DISEASES

**FIGURE 1:**
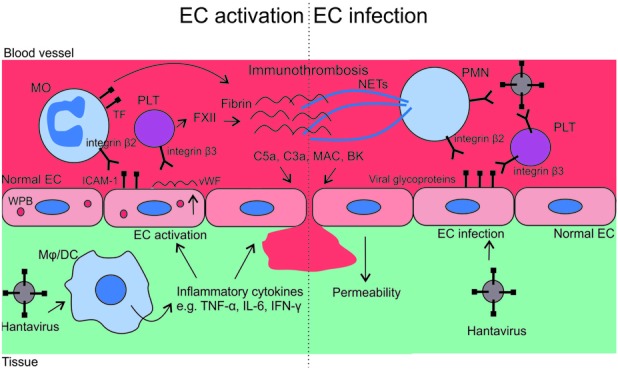
**Mechanisms of vasculopathy in hantavirus infections.** The recognition of hantaviruses by macrophages (Mφ) or dendritic cells (DCs) induces proinflammatory cytokines, which evoke a change from anti- to pro-adhesive phenotype of endothelial cells (ECs). Pro-adhesive ECs bind monocytes (MOs) through ICAM-1 – integrin β_2_ interaction, and platelets (PLTs) through vWF through α_IIb_β_3_ integrin interaction. Activated MOs and PLTs then respectively promote coagulation through tissue factor (TF) and contact activation pathway (factor XII), to restrict the spread of the virus. Simultaneously hantavirus-infected ECs display viral glycoproteins on their surface, which respectively bind β_2_ and β_3_ integrins of polymorphonuclear neutrophils (PMNs) and PLTs. The binding results in the release of neutrophil extracellular traps (NETs) from PMNs and increased activation of PLTs. These virus-induced events enhance inflammation and may result in an excessive formation of immunothrombosis. Complement and contact pathway activations, both associated with immunothrombosis, contribute to vascular leakage through anaphylatoxins C5a and C3a, membrane attack complex (MAC) and bradykinin (BK).

Plasma leakage from vasculature into tissues is a hallmark of hantavirus infection. Clinically, this is presented by hemorrhages (the presence of plasma fluid in tissues), hemoconcentration (relative cell number increase in plasma), and hypotension (decreased blood pressure). Vascular leakage can be caused by either enhanced endothelial cell (EC) permeability or by direct injury to the vasculature. In HFRS, widespread EC swelling, perivascular edema, diapedesis of erythrocytes, and mononuclear cell infiltrates without evidence of EC damage have been observed by microscopy (Tsai, 1987). This suggests that endothelial barrier function is lost due to enhanced permeability rather than by direct cellular cytotoxicity or injury of the vasculature. Hantavirus antigens are present in ECs during HFRS (Cosgriff, 1991) and in ECs of lung capillary during HCPS (Zaki et al., 1995), but based on *in vitro* studies hantavirus infection of ECs does not induce direct cytopathic effects (Yanagihara and Silverman, 1990; Pensiero et al., 1992; Valbuena and Walker, 2006; Mackow and Gavrilovskaya, 2009; Vaheri et al., 2013). However, virus-induced general inflammation may compromise the barrier function of the endothelium and induce vascular leakage. If so, similar mechanisms could be behind the hemorrhages seen in other viral infections. On the other hand, the infection of ECs might lead to virus-specific promotion of permeability. Evidence in favor for both scenarios is discussed in the following paragraphs. Hypotheses on increased vascular permeability in hantavirus diseases are presented in **Figure 1**.

## INFLAMMATION

Endothelial cell activation occurs in HFRS. Upregulated levels of soluble EC receptors: E-Selectin (Takala et al., 2000), intercellular adhesion molecule (ICAM; Han et al., 2010), and tumor necrosis factor receptor (TNFR)-1 (Kyriakidis and Papa, 2013) are released into circulation during acute HFRS. While there is no evidence on EC activation in HCPS, the upregulation of pro-inflammatory cytokines: interleukin (IL)-6, tumor necrosis factor (TNF)-α, and interferon (IFN-γ) that all are capable of activating the endothelium, have been reported in both hantavirus diseases (Linderholm et al., 1996; Peters et al., 1999; Klingstrom et al., 2002; Abel Borges and Figueiredo, 2008; Sadeghi et al., 2011; Saksida et al., 2011; Korva et al., 2013; Kyriakidis and Papa, 2013). Especially high-levels of TNF-α is linked with a more severe disease (Kanerva et al., 1998; Makela et al., 2001; Borges et al., 2010; Korva et al., 2013). Pro-inflammatory cytokines are mainly produced by activated macrophages. It is known that macrophages can be infected by hantavirus which could lead to their activation (Vaheri et al., 2013). These cytokines induce EC permeability either directly or via EC activation, which leads to leukocyte recruitment and subsequent EC gap formation (Marcos-Ramiro et al., 2014). Leukocytosis is common in hantavirus diseases and probably relates to the inflammatory response against the pathogen. Interestingly, a recent report indicated that neutrophil activation through extracellular trap formation occurs in the mild form of HFRS (Raftery et al., 2014). Hantaviruses can activate neutrophils *in vitro* by direct binding but also pro-inflammatory ECs will recruit neutrophils. The role of pro- or anti-inflammatory response to hantavirus infection in the rodent host respectively promote either viral clearance or tolerance (Easterbrook and Klein, 2008; Guivier et al., 2010; Li and Klein, 2012). Therefore, it seems that while the pro-inflammatory response of the host is required for virus clearance, its excessive activation will lead to EC permeability and subsequent vascular leakage.

## COMPLEMENT ACTIVATION

Complement activation occurs in acute HFRS, as judged by decreased C3 and increased membrane attack complex (MAC) levels in plasma (Guang et al., 1989; Paakkala et al., 2000; Sane et al., 2012; Laine et al., 2014), and it correlates with upregulation of pro-inflammatory cytokines and disease severity (Laine et al., 2014). Complement activation produces circulating anaphylatoxins, C3a and C5a, which may cause EC activation and permeability in addition to direct MAC-mediated vascular injury (Kerr and Richards, 2012). Complement activation could be inflammation-dependent (Takano et al., 2013). However, complement might be activated also by virus-related immune complexes that are seen on the surface of ECs and platelets in HFRS (Penttinen et al., 1981; Guang et al., 1989). We recently reported upregulation of galectin-3 binding protein (Gal-3BP) in acute HFRS, and found a correlation between Gal-3BP and MAC levels (Hepojoki et al., 2014). We also demonstrated that hantavirus infection induces Gal-3BP production in ECs (Hepojoki et al., 2014), and such overproduction could sensitize the infected cells for complement attack. Furthermore, our unpublished data show that Gal-3BP interacts with hantavirus particle, and thus also the binding of Gal-3BP to either virions or to the surface of infected cells may promote complement activation. Glomerular ECs are the prime site of complement attack (Takano et al., 2013). Interestingly, we found that Gal-3BP is produced in the glomeruli and tubular epithelium of PUUV-infected macaques. The complement attack against glomerular EC could contribute to kidney dysfunction in hantavirus diseases. Decay-accelerating factor (DAF or CD55) acts as a controller of complement activation on cell surfaces. Curiously, DAF also interacts with both New and Old World hantaviruses (Krautkramer and Zeier, 2008; Buranda et al., 2010; Popugaeva et al., 2012), and the interaction might affect DAFs physiological functions resulting in increased complement activation. On the other hand, hantavirus infection of renal glomerular (e.g., podocytes) and tubular cells results in disruption of cell-cell contacts that could directly lead to decreased kidney barrier function and subsequent proteinuria (Krautkramer et al., 2011). Also, soluble urokinase-type plasminogen activator receptor (suPAR), elevated in the plasma and urine of HFRS patients, could affect podocyte integrity (Outinen et al., 2013, 2014).

## IMPAIRED HEMOSTASIS

Increased coagulation is associated with hemorrhages especially in HFRS. Laboratory findings such as increased bleeding time, prothrombin time, activated partial thromboplastin time, and thrombin time together with decreased plasma activity of several coagulation factors, and the presence of fibrin degradation products are indicative of DIC in severe HFRS (Guang et al., 1989; Lee et al., 1989). The decreased levels of coagulation factors compromise the barrier function of vasculature and lead to increased bleeding times. Additionally, the increased activity of thrombin can directly induce EC permeability (Kleinegris et al., 2012). Both increased coagulation and fibrinolysis are present also in the mild form of HFRS, even though hemorrhages are not commonly observed (Lahdevirta, 1989; Laine et al., 2010, 2011, 2014). Although coagulation abnormalities are recognized in HCPS (Duchin et al., 1994), they have not been comprehensively studied. Given the central role of platelets in coagulation, it is likely that thrombocytopenia in hantavirus diseases is due to increased peripheral consumption. On the other hand, the loss of platelets from circulation could be due to platelet binding of infected ECs as suggested by *in vitro* studies (Gavrilovskaya et al., 2010).

Extrinsic and contact system pathways can induce coagulation. Increased activity of plasma kallikrein in HFRS patients (Guang et al., 1989) is suggestive of contact system activation. Corroborating this notion, one severely ill patient with NE was successfully treated with icatibant, a bradykinin receptor antagonist (Antonen et al., 2013; Vaheri et al., 2014). Bradykinin is a peptide produced in plasma through kallikrein–kinin system and it promotes vascular permeability. Furthermore, the surface of hantavirus-infected ECs promotes kallikrein activation, bradykinin formation, and increased permeability when incubated with proteins involved in the kallikrein–kinin pathway of plasma (Taylor et al., 2013). A shift from anticoagulant to procoagulant-state is seen in the endothelium of HFRS patients. In acute HFRS von Willebrand factor (vWF) and coagulation factor VIII, normally residing in Weibel–Palade bodies of ECs (Rondaij et al., 2006), are released in to the circulation (Guang et al., 1989; Laine et al., 2011). The exocytosis of Weibel–Palade bodies is further corroborated by the detection of increased levels of angiopoetin-2, a protein promoting vascular permeability (Eklund and Saharinen, 2013) in HFRS (Krautkramer et al., 2014).

The activation of ECs together with complement and coagulation pathways in hantavirus diseases is suggestive of immunothrombosis (Engelmann and Massberg, 2013), which is a form of innate immunity that acts by trapping blood-borne pathogens to a “mesh” of fibrin and chromatin. Fibrin is a product of thrombin activity and extracellular chromatin is released from activated neutrophils and monocytes. Failure of immunothrombosis to restrict the spread of the virus may trigger DIC via unrestricted formation of microvessel thrombi and the excessive activation of inflammation. Immunothrombosis could thus represent the first physiological stage in the development of severe hantavirus disease.

## CYTOTOXIC T CELLS AND HUMORAL IMMUNE RESPONSE

The possible role of the cytotoxic T cells (CTLs, also referred to as CD8^+^ T cells) in hantavirus pathogenesis is extensively reviewed elsewhere (Terajima and Ennis, 2011). It is clear that CTLs are upregulated in both acute HFRS and HCPS (Huang et al., 1994; Kilpatrick et al., 2004; Wang et al., 2009; Lindgren et al., 2011; Rasmuson et al., 2011). One mechanism on how CTLs might enhance vascular permeability is direct killing of hantavirus-antigen positive ECs. However, cell death is not obvious in patients. Lately, hantavirus-infected ECs were found to block CTL and natural killer (NK) cell cytotoxicity *in vitro* (Gupta et al., 2013), thus providing an explanation for the discrepancy. Despite this, CTLs as well as other hematopoietic cells may contribute to the increased EC permeability by releasing pro-inflammatory cytokines. The upregulation of Gal-3BP, a potent stimulator of CTLs and NK cells (Ullrich et al., 1994), in acute hantavirus infection (Hepojoki et al., 2014) might play a role in the pathogenesis of hantavirus disease.

Hantavirus infection also induces a strong antibody response against the structural proteins of the virus (Vapalahti et al., 1995, 2001). Curiously, the appearance of antibodies against the viral proteins coincides with the occurrence of symptoms. Also rheumatoid factor (RF) is present at the same time (Penttinen et al., 1981). Both RF and antibodies could contribute to complement activation, which in turn could compromise vasculature’s barrier functions.

## INFECTION OF ECs

Pro-inflammatory cytokines and mediators of both complement and coagulation cascades are mainly produced by activated monocyte/macrophages or cleaved from plasma proteins. But what is the role of replication in ECs for hantavirus pathology? Hantavirus infection of ECs induces interferon (IFN-β) and chemokines RANTES (regulated on activation, normal T cell expressed and secreted) and IP-10 (IFN-γ inducible protein) *in vitro* (Sundstrom et al., 2001; Geimonen et al., 2003; Kraus et al., 2004; Khaiboullina et al., 2013). However, the majority of reports indicate that hantavirus infection *per se* does not alter EC permeability (Yanagihara and Silverman, 1990; Pensiero et al., 1992; Khaiboullina et al., 2000; Sundstrom et al., 2001; Gavrilovskaya et al., 2008), although vascular endothelial growth factor (VEGF)-dependent permeability increase occurs in ANDV infection (Shrivastava-Ranjan et al., 2010). Interestingly, there is a decrease in the level of the tight junction protein ZO-1 in HTNV-infected glomerular ECs that likely affects the barrier function of glomerulus (Krautkramer et al., 2011). Except for innate immunity activation, very little data supports EC activation in response to hantavirus infection *in vitro*, suggesting that EC infection would not contribute to inflammation.

It seems widely accepted that pathogenic hantaviruses differ from non-pathogenic viruses by their ability to delay early innate immunity induction (i.e., IFN-β), which would selectively restrict replication of apathogenic viruses (Geimonen et al., 2003; Spiropoulou et al., 2007; Matthys and Mackow, 2012). These observations are mainly based on PHV and could in the future be complemented by studies with other apathogenic or low-virulent hantaviruses. This would be very interesting, since even different isolates of the same virus markedly differ in replication kinetics and in recognition by the innate immunity machinery (Sundstrom et al., 2011). Integrins have been, according to *in vitro* studies, declared as the cellular receptors of hantaviruses, and PHV is distinct from all pathogenic hantaviruses studied in its ability to use α_5_β_1_ instead of α_V_β_3_ integrin on ECs (Mackow and Gavrilovskaya, 2009). However, since the virulence of HFRS- and HCPS-causing hantaviruses (both claimed to use β_3_-integrin) differs dramatically, the role of integrin-receptor on virulence is scanty. In fact Sangassou hantavirus, capable of infecting humans (Klempa et al., 2010), was shown prefer β1 integrins for entry *in vitro* (Klempa et al., 2012). Integrins of hematopoietic cells (platelets and neutrophils), interact *in vitro* with hantaviruses to mediate adherence of platelets on infected ECs and release of chromatin from neutrophils (Gavrilovskaya et al., 2010; Raftery et al., 2014). Such interactions could directly contribute to thrombocytopenia, coagulopathy and EC permeability.

Endothelial cell infection could lead to viremia, which has been postulated to play an important role in the vascular dysfunction (positive correlation with hemoconcentration and thrombocytopenia) and worsened disease outcome in SNV-caused HCPS (Terajima et al., 1999). In concordance, more viral RNA has been found in plasma of patients with DOBV (severe HFRS) as compared to PUUV (mild HFRS; Korva et al., 2013). Similarly, more DOBV than PUUV is found in their respective carrier rodents (Korva et al., 2009). These findings suggest, possibly trivially, that the actual amount of infecting virus could be an important factor for pathogenesis. This is also supported by *in vitro* infections where the initial viral load negatively correlates with cell survival (Strandin et al., 2008).

## VASCULAR ENDOTHELIAL GROWTH FACTOR

Vascular endothelial growth factor induces angiogenesis, which is accompanied by increase in vascular permeability. *In vitro* observations show that hantavirus infection renders ECs hypersensitive to the permeabilizing effects of VEGF and VEGF levels are increased in both HFRS and HCPS (Shrivastava-Ranjan et al., 2010; Gavrilovskaya et al., 2012; Ma et al., 2012; Tsergouli and Papa, 2013; Krautkramer et al., 2014), but at different kinetics. While in HCPS the VEGF levels return to normal in the recovery phase, the VEGF level may remain high in HFRS from the febrile to early convalescent phase. This suggests that VEGF would not contribute to disease development in HFRS, but would rather mediate angiogenesis and vasculature repair in the recovery. Increased levels of circulating endothelial progenitor cells (EPCs), correlating with disease recovery, are observed in NE (Krautkramer et al., 2014).

## CONCLUSION

Human hantavirus infection is a dead-end for the virus. Humans and the different reservoir hosts differ genetically, and the genetic differences in receptors, and in the mediators of immune response likely contribute to the course of the disease. The degree of homology and molecular mimicry between the reservoir host and human might partially explain the varying degree in disease severity between different hantaviruses. The same factors could also explain the differences in pathogenesis between different hantaviruses. All human hantaviruses initially enter the lung. The HCPS causing hantaviruses predominantly cause the disease already in the lung, whereas HFRS causing hantaviruses find their way into kidneys. The degree of disease severity is also affected by individual differences in for instance immune activation. In overall it seems that there are several simultaneously occurring factors, which contribute to the permeability increase, in hantavirus infection.

## Conflict of Interest Statement

The authors declare that the research was conducted in the absence of any commercial or financial relationships that could be construed as a potential conflict of interest.
